# Associative Memory Storage and Retrieval: Involvement of Theta Oscillations in Hippocampal Information Processing

**DOI:** 10.1155/2011/683961

**Published:** 2011-09-26

**Authors:** Federico Stella, Alessandro Treves

**Affiliations:** ^1^SISSA, Cognitive Neuroscience Sector, 34136 Trieste, Italy; ^2^Kavli Institute for Systems Neuroscience and Centre for the Biology of Memory, NTNU, 7030 Trondheim, Norway

## Abstract

Theta oscillations are thought to play a critical role in neuronal information processing, especially in the hippocampal region, where their presence is particularly salient. A detailed description of theta dynamics in this region has revealed not only a consortium of layer-specific theta dipoles, but also within-layer differences in the expression of theta. This complex and articulated arrangement of current flows is reflected in the way neuronal firing is modulated in time. Several models have proposed that these different theta modulators flexibly coordinate hippocampal regions, to support associative memory formation and retrieval. Here, we summarily review different approaches related to this issue and we describe a mechanism, based on experimental and simulation results, for memory retrieval in CA3 involving theta modulation.

## 1. Introduction

Theta-like oscillations are widely spread across the brain, particularly of rodents, and modulation in this frequency range can be found in different brain regions [1]. Theta oscillations are an exceptionally appealing subject for modeling, due to the wealth of suggestive experimental data, ranging from behavioral to single-cell recording. These data show on the one hand that the influence the theta rhythm exerts on neuronal activity is significant and on the other hand that its presence correlates with the performance of cognitive tasks. The question, of course, is how to describe mechanistically the link between these two aspects. Understanding the role of theta oscillations in hippocampal processing requires reviewing the potential contribution of oscillations to neural computation.

The ubiquitous nature of theta-range oscillations suggests that they can be used by the brain to coordinate different areas and to manage the communication and the flow of information among them [2–7]. This perspective emphasizes the need for a coordination mechanism to make the brain work properly [8–10]. Any cognitive task requires the involvement not only of a number of distinct brain regions, but also of different subnetworks within the same regions, which are not physical adjacent and can utilize only limited direct connections. Each of these networks operates on the input it receives and feeds its output to other networks, which in turn do the same. To successfully deal with the outside world, sensory input has in general to be processed many times. Moreover, these processes have to be carried out in a certain sequence and with certain timings. An oscillatory modulation of network activity, involving several brain structures and synchronizing the discharge times of neurons, seems like a good candidate to orchestrate these intricate processes. From this perspective, theta rhythm does not have a specific functional role in the operations performed by a particular brain region, rather it is used to globally coordinate activity [11, 12].

Another perspective, which does not necessarily exclude the former, is that these oscillations are instead essential to the specific operations performed by the various local networks of the brain. The way information is organized may depend on the way the theta rhythm induces neurons to fire. The modulation can induce particular patterns of firing in a population. In this perspective, the oscillations are crucial to explain how a certain brain region processes the information it receives and to understand the features of the representations that are generated. 

In this paper, we shall review some ideas about a specific role of theta oscillations in the hippocampus and then present and discuss a mechanistic model, inferred from recent experimental observations, of how theta modulation could augment an independently existing hippocampal function. Rather than a special case, the hippocampus is emblematic of the use of oscillations in neural theories [4, 13, 14]. This is because the theta rhythm is particularly strong in this region, in rodents, and it strongly modulates its activity [15]. Also, most of the ideas about theta function were first inspired by observations in the rodent hippocampus.

## 2. Theta Oscillations in the Rodent Hippocampus

The hippocampal theta rhythm is a most prominent clocking mechanism in the forebrain [15]. The rhythm, in the 5–10 Hz band in the rat, can be readily detected as a macroscopic local field potential (LFP) in the dorsal hippocampus during exploratory behavior and REM sleep [9, 16]. According to the simplest model of theta generation, the medial septum in the basal forebrain functions as a pacemaker, enforcing a global rhythm into which hippocampal and entorhinal cortex (EC) networks are entrained. However, evidence obtained with numerous experimental manipulations indicates that several rhythm-generating mechanisms and theta current dipoles are independently at work [17–19]. The idea of a coherent wave of activity turning hippocampal neurons periodically on and off, as a single entity, has faded with the discovery of a much more complex pattern of activation of the various hippocampal subpopulations. 

### 2.1. Not One Theta Oscillator but Many

**Figure 1:**
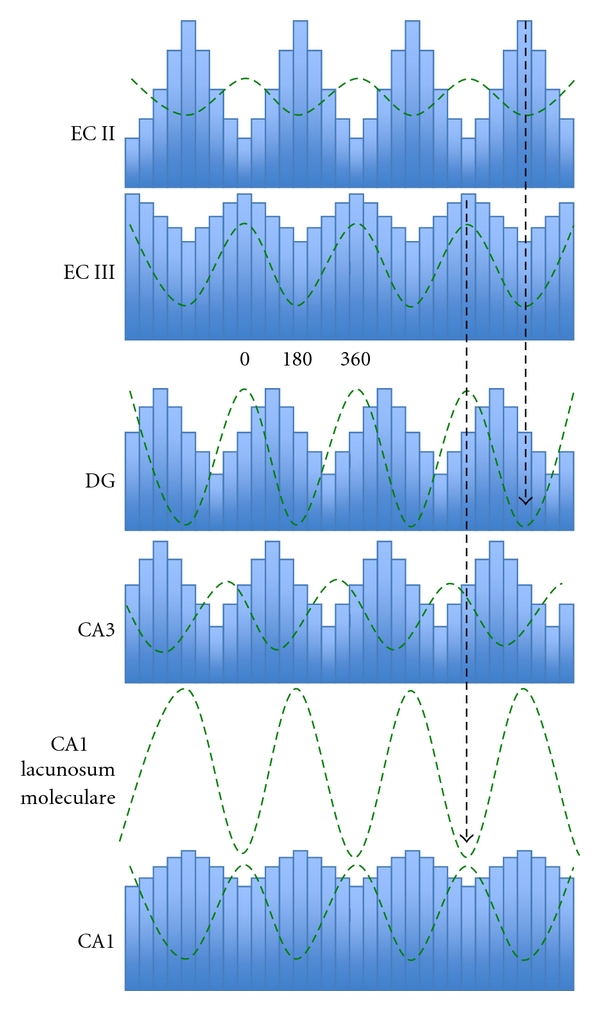
Schematic firing rate modulation in hippocampal and parahippocampal regions. The firing phase of principal cells is indicated by the histograms, while the curves describe LFP amplitudes at different locations (see text). Redrawn from [17].

The hippocampus is not passively responding to a single theta generator. Several studies [9, 19] have shown that each lamina of the hippocampal formation acts as a distinct oscillator, with its own theta rhythm. Although these multiple oscillators differ in phase and amplitude, they generally present the same frequency [17]. The uniformity in the oscillation frequency across different layers nevertheless points at the influence of an external common pacemaker, generally identified in the medial septal area. Indeed, the inactivation of this area, or the interruption of its projection to the hippocampus, has a dramatic effect on theta dynamics in the region [20]. The LFP theta rhythm, along with rhythmic hippocampal unit activity, is eliminated, together with any coherence among different regions of the hippocampal formation. In normal conditions, sharing the same frequency but at different phases results in the appearance of particular coordination profiles among these regions (Figure 1). Recorded LFPs show increasing and decreasing intensity with characteristic inter-layer intervals, which remain more or less constant across time. More precisely, the two most prominent theta dipoles, the one in the stratum lacunosum moleculare of CA1 and the other in the stratum moleculare of the DG, alternate in phase, while the current-source density in other layers shows intermediate phase relations. The theta modulation recorded in entorhinal cortex, which has been demonstrated to be at least partly independent [21–23], shows an inversion in phase between layer I and deeper layers. Layers II and III, where most of the projections to the hippocampus originate, thus oscillate in phase, and this phase is the same as the one typical of the dipole located in the stratum lacunosum moleculare of CA1.

When examining the amplitude, frequency, phase, and coherence relationships among these dipoles during various aspects of behavior, however, it has been found that the power, the coherence, and the phase of theta oscillations exhibit layer-specific changes that depend on behavioral task demands [19, 24]. As a consequence, the relations among the timing of theta oscillations in the various hippocampal layers can be modified by the particular cognitive state of the animal. Because the different layers are associated with particular physiological structures, such as distal and proximal dendrites and cell bodies, these modifications can influence the way spiking activity in the various layers is generated. This implies that oscillations can support specific processes, necessary only in certain conditions. A recent study [25] has complicated this situation even further, by indicating that theta oscillations are not synchronized within stratum oriens of CA1 but are traveling waves that propagate predominantly along the septotemporal hippocampal axis. This study is in accordance with the finding that theta dynamics are not homogeneous along the same axis: theta power and the fraction of theta-rhythmic neurons are reduced in the ventral portion of CA3 compared to the dorsal portion [26, 27]. Again, this inhomogeneity has been hypothesized to enable specific computational functions [28, 29]. 

This body of experimental evidence indicates that theta rhythm is not a monolithic clock signal: hippocampal theta oscillations interact with a heterogeneous consortium of transmembrane currents, reflecting layer-specific processing that can be modulated by extrahippocampal inputs or differential modes of operation within the hippocampus.

### 2.2. Influence on Firing Rates

In a network of synchronized neurons, the oscillatory phase determines the degree of excitability of the neurons. In which way do hippocampal populations discharge with respect to the theta phase? We expect the maximal activity of neurons to be coincident with what is conventionally denoted as the trough of theta, when the membrane is depolarized. And because of the variety and heterogeneity of theta oscillations, we expect that neurons belonging to different subregions should fire at different times, in accordance with the local dipole. This is found to be only partially true [17]. Indeed, the activity of all the neurons of the hippocampus is modulated, to different degrees, by the theta rhythm. The strength of this modulation is not defined by the amplitude of the corresponding theta oscillation, however. CA3 neurons show the strongest modulation, while CA1 modulation is significantly less pronounced, even if the strongest theta dipole is in the CA1 region. The preferred phase does not necessarily correspond to the theta trough, either. So, for example, the neurons of EC layers II and III fire at opposite phases of the common reference oscillation, layer II neurons at the trough, and layer III neurons at the peak (Figure 1). This results in the fact that while the peak of population activity in an upstream region may match the timing of dendritic excitation of downstream target neurons (current sinks) in the hippocampus, the discharge of the respective target populations could be offset of even half a theta cycle. Also interneurons show the effects of the theta modulation [30, 31]. As pyramidal neurons do, also the different classes of interneurons have phase preferences. Different types have different preferred phases [32–36]. Interestingly, it appears that dendritic targeting interneurons tend to group together and to have the same discharge phase, different from the one preferred by somatic targeting interneurons, similarly grouped on their own [37].

The local theta does not seem to constrain the discharge to occur at its trough. Each population has a preferred phase of its local theta oscillation. And if we take a global look at the distribution of spikes, taking as a reference a single theta oscillator, we see that different regions discharge with specific times with respect to others, generating a complex sequence of activation connecting the various parts of the circuit [17–19]. This pattern does not need to be the same at all times. Also the preferred phase of certain populations shifts according to the task [24]. 

### 2.3. Theta Oscillations and Information Processing in the Hippocampus

A most intriguing question about the theta rhythm, which has not been answered, is its link to information processing in the hippocampus. Not only it appears that the function of this region is persistently modulated during most awake activity, but also we have seen that the modulation is finely articulated through the different regions of the hippocampus. The role of these oscillations in the operations performed by the hippocampus might then be indicated by the relative timing at which different populations oscillate.

Work on the rat hippocampus suggests at least three possible functions for theta [38].

- First, it may act as a global synchronizing mechanism, locking the entire hippocampal formation into one global processing mode and organizing the activity in each hippocampal region with respect to the rest. This means that if two cells have firing patterns that are systematically related to the local theta cycle, they have systematic temporal relations to each other, even if they are located far apart in the hippocampus.
- A second possible function for theta is to provide control over long-term potentiation induction [39–41]. This perspective takes into account evidence that synaptic transmission and plasticity are differentially effective depending on the phase of theta. Inputs arriving at the positive phase of theta in the dendritic layer are more likely to lead to synaptic potentiation, whereas those arriving at the negative phase yield more often depotentiation or depression.
- A third putative function of theta oscillations could be to provide a periodic clock for the timing of hippocampal spikes, acting like a temporal reference frame for any activity in the region [42, 43]. This hypothesis is based on the observation that the phase of firing of each pyramidal cell, relative to theta, is not constant but can vary from one cycle to the next. As a rat runs through the firing field of a pyramidal cell, its place field, the cell fires bursts of spikes at a frequency slightly higher than that of the concomitant theta. This leads to the so-called phase precession phenomenon, which is the tendency of the cell to fire at earlier points of a theta cycle on each successive cycle [44]. The processing phase is thought to express spatial information in addition to that expressed by the firing rates alone [45–47]. In fact, the particular sequence of spikes in each theta cycle may be associated to a particular location and local trajectory in the environment and therefore presumed to carry detailed spatial information.

In the following we will discuss theories representative of the first two categories. Phase precession has been extensively studied, debated, and reviewed elsewhere, and we will focus on models that deal more with the modulatory character of the theta rhythm [38].

## 3. Storage and Retrieval

How are the memory functions of hippocampal circuits enhanced (or interfered with) by oscillations of physiological variables in the theta frequency range? The presence of oscillations in the hippocampus and their persistence in a wide range of different cognitive states suggest the possibility that this periodic modulation of neural activity might have a major role in memory processes.

### 3.1. The Acetylcholine Hypothesis

A generic problem with associative memories based on recurrent connections is distinguishing a storage mode from a retrieval mode. To be effective, recurrent connections should dominate the dynamics of the system when it is operating in retrieval mode; whereas while storing new information the dynamics should be primarily determined by afferent inputs, with limited interference from the memories already stored in the recurrent connections, which instead should modify their weights to store the new information. A possible solution to implement the dual operating mode is to use a modulator that acts differentially on the afferent inputs, that are located at the apical dendrites, and on the recurrent connections, lower on the dendritic tree. Acetylcholine can achieve this effect, exploiting the orderly arrangement of pyramidal cell dendrites in the cortex [48–50]. It causes differential physiological effects on the different synaptic connections of cortical neurons. Activation of muscarinic acetylcholine receptors enhances the rate of synaptic modification at excitatory feedback (including recurrent) connections, as seen in experiments showing cholinergic enhancement of long-term potentiation. At the same time as it enhances long-term potentiation, acetylcholine suppresses excitatory synaptic transmission at feedback synapses, while leaving excitatory feedforward synapses relatively unaffected. Thus, feedback synapses are weak in the presence of acetylcholine, but still their activation leads to enhanced LTP, which makes them stronger at later times. This combination of effects can be very useful to encode new information. Effective formation of associative memories requires that network activity be defined by feedforward input during encoding. This prevents new associations from being distorted by the spread of activity across previously modified feedback connections. Thus acetylcholine may prevent interference during the strengthening of feedback synapses by selectively suppressing excitatory feedback synapses but not feedforward synapses.

Even if acetylcholine has a major influence on synaptic plasticity, this mechanism presents two limitations. First, it requires an active process that distinguishes storage from retrieval periods and regulates acetylcholine release accordingly. Modulation of network dynamics through acetylcholine requires a top-down control agent that actually takes the decision of switching the system from an encoding state to a retrieving one and vice versa. Further, the muscarinic cholinergic effect on synaptic transmission and postsynaptic depolarization has a relatively slow time course. Acetylcholine would require several seconds to change network dynamics [51]. This has motivated a search for alternative or additional mechanisms for separating encoding from retrieval. Faster transitions between the two modalities and independence from an external controller would be the ideal requisites for these alternative mechanisms. 

Hasselmo et al. [52] have proposed, based on evidence concerning LTP induction during theta modulated activity, that the various phases of theta oscillation themselves represent different modes of operation, that is, storage and retrieval.

### 3.2. The Hasselmo Model

**Figure 2:**
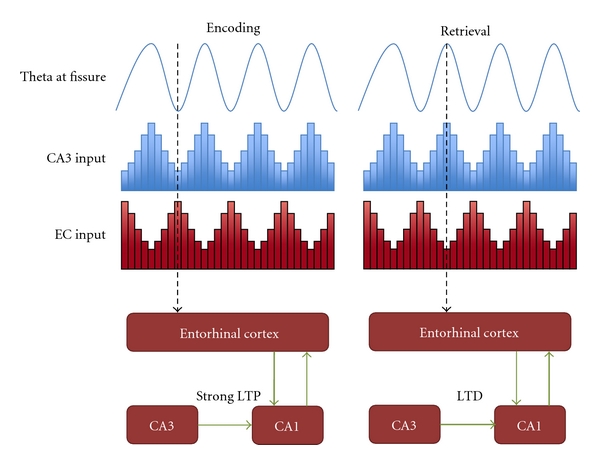
Schematic illustration of the Hasselmo et al. model. Redrawn from [52].

The model proposed by Hasselmo and others in a number of papers offers a possible solution to the problem [52–54]. It focuses on CA1. The issue it tries to address is how the dynamics of this region can distinguish between a phase in which the inputs coming from CA3 should lead to synaptic modification of the Schaffer collaterals and another phase in which the inputs coming from the very same connections should trigger pattern retrieval, this time without changing Schaffer collaterals strength. Hasselmo's proposal is to use the theta rhythm and to segregate encoding and retrieval into different phases of the oscillations (see Figure 2). He bases his model on the following evidence.

- First, the laminar organization of theta oscillations. The strongest inputs from layer III of entorhinal cortex, which projects to stratum lacunosum-moleculare through the perforant path (PP), come when there are prominent current sinks in the target layer, at the trough of the local LFP. The idea is that this strong depolarizing input to the distal dendrites could allow encoding even in the absence of spiking activity of the neurons in stratum pyramidale, where strong inhibition due to outward currents acts on the cell bodies. As the spiking activity is so reduced, no spurious retrieval of previous memories should be possible at this Θ-trough phase. This is then proposed as the encoding phase. In contrast, because of the inversion of theta polarity between stratum lacunosum-moleculare and stratum pyramidale, at the peak of the LFP in the former there is a prominent trough in the latter, and also in stratum radiatum. At this Θ-peak the activity of CA1 neurons is maximal, and retrieval should take place at this time.
- Second, the separation of encoding and retrieval in this model depends upon phasic changes in the efficacy of LTP induction. After a number of studies in slices or in anesthetized animals, recently there have been some results also in awake, freely moving rats, which all show an interaction between synaptic plasticity and theta rhythm [39–41]. It appears in fact that LTP is induced best when stimulation reaches the dendrites at the peak of theta in the corresponding dendritic layer. The induction of LTP in stratum radiatum, then, where the Schaffer collaterals make their connections with CA1 cells dendrites, would largely occur when transmission is weak but dendrites are depolarized by entorhinal inputs arriving higher up on the dendritic tree, in stratum lacunosum-moleculare. It appears that LTP does not require somatic spiking in the postsynaptic neuron, as dendritic spikes can induce LTP even when the soma is hyperpolarized [55].

Network simulations of this system, intended to reproduce the physiological data of EC layer III, CA3, and CA1, have been used to direct the movements of a virtual rat in a virtual environment in tasks including spatial reversal, spatial alternation, and delayed nonmatch to position [52, 54, 56]. A mathematical analysis of the performance of the rat was then used to determine which phase relations were optimal. It was found, in accordance with real data, that the phase of synaptic input from CA3 should match the phase of maximal depolarization at CA1 cells bodies, and it should be 180° out of phase with EC input. The authors propose that their model can account for behavioral data showing that fornix lesions (which reduce theta power) cause an increase in the number of errors after reversal in a T-maze task. Rats with fornix lesions persist in visiting the arm that was previously rewarded, even when it is unrewarded. The claim is that this behavior is caused by the reduced theta rhythm and by the consequently reduced separation, in stratum radiatum, between LTP and synaptic transmission, which are now strong at the same time. What happens is that, after reversal, the rat makes erroneous visits to the wrong, previously rewarded arm, guided by the learned rule. Strong synaptic transmission induces the hippocampus to retrieve a pattern in CA1 that corresponds to past memory of food in the now unrewarded location. Moreover, this retrieval activity, in the absence of segregation between the two functions, can cause further LTP, thus strengthening the association with the memory of food, even if now the location is unrewarded. This mechanism could therefore slow down the extinction of old associative memories, delaying reversal learning and increasing the number of errors.

A weak point of this theory is that it is not clear how strong associations between the activity in CA3 and in CA1 can be acquired when very few cells are firing. Even if, in the case of the cells in CA1, the depolarization caused by EC inputs may be sufficient for plasticity, without spiking, the model still needs action potentials generated by cells in CA3. The storage phase should take place at times in which the activity in CA3 is minimal. If only a small subset of the neurons in CA3 is firing, only a small part of the full representation is active and can thus be transferred to CA1. Most of the cells that participate in retrieval did not fire during encoding and could not modify their synapses with CA1 cells.

## 4. The Case of CA3

The model above is a valid attempt to deal with a problem which looms upon any theory of memory formation, and it has the merit of shifting the search for a possible solution from the action of a specific neuromodulator to the organization of the internal dynamics of the system. It appears, however, that its applicability is restricted to CA1, whereas the problem of separating the two regimes affects the upstream region CA3 as well. In fact, in CA1 we may see only the reflection of a conflict which originates in CA3. After all, it is there that most memory storage might take place, according to a mainstream view, and it is starting from there that memory is retrieved. CA1 may largely be a mere relay of a signal originated in CA3 [57–61]. Although recent studies point to a more complex interaction between this signal and the one coming from EC and converging in CA1 [62, 63], CA3 processes seem to be the crucial ones for the memory functions of the system [64, 65]. 

### 4.1. The Idea of the Dentate Gyrus Input as a “Detonator” for CA3 Activity

Comparative neuroanatomy suggests [66] that mammals may have evolved a more refined mechanism to separate storage from retrieval, to efficiently perform both functions in a passive mode, by inserting a preprocessor before the CA3 memory network [67]. The preprocessor would instruct which units in CA3 should fire when a new distribution of activity is stored as the memory representation of a new item to be remembered. This preprocessing network forms new arbitrarily determined representations on the fly and through a system of strong connections (“detonator” synapses) [68] imposes these new representations onto CA3. The key ingredients that make this machinery work and allow new representations to form, providing them with meaningful content, against the interference of recurrent connections, are simply sparse and strong connections from a sparsely coded feed-forward network [69, 70]. Because the representation in the preprocessor is effectively randomly generated, as the connections from the preprocessor to CA3 also are, the representations of different memories that this mechanism will impose in CA3 tend to be decorrelated [71]. The challenge for afferent input is to prevail over the recurrent connections, which do not impart new contents to a pattern of activity to be stored. This challenge can be met by afferent inputs with the characteristics of the mossy fibers [72], but not by those with the characteristics of the perforant path to CA3. The idea thus is to avoid a conflict between encoding and retrieval by maximizing the distance among distinct memories. Recruiting different subsets of neurons to participate in the representations of different items would be the main role of the mossy fibers. The point here is that moderately similar inputs, thanks to the operation of the intermediate processing stage represented by the DG, are represented in CA3 by patterns of activity much less correlated than the inputs were [58], and if two inputs are so similar as to elicit the same representation, it may imply that they are actually the same thing [57], at least from the point of view of a memory network like the hippocampus. 

With this mechanism, storage and retrieval may be continuously interleaved, as part of the same process initiated by the DG. As the firing activity between the two regions is almost synchronized, the communication is maximal. The representation elicited in DG contributes in its entirety to define the activity in CA3 during storage, when the recurrent collaterals are modified, but also in a partial reactivation during retrieval, which can take place based on a partial cue. The other element of this mechanism is the perforant path, projecting from EC layer II to CA3. Because of their weakness, these connections are not presumed to play a relevant role during storage, while during retrieval with their associatively modified synapses they can relay most effectively the cue that initiates retrieval. 

In this framework, the role of theta oscillations appears to be ancillary. They may help in coordinating information flow between DG and CA3, and then CA1, but their presence is not functionally essential, at least for what concerns the segregation of storage and retrieval. 

This solution to the storage/retrieval conflict possibly extends also to CA1. Moreover, even if the region receives largely decorrelated inputs, which should thus produce equivalently distinct representations in CA1, the mechanism proposed by Hasselmo et al. [52] may still enhance the decorrelation, as well as possibly serving other purposes, for example, to modify, in accordance with direct PP input, the representations induced by CA3, in order to recover some of the information dissipated during the storage process.

## 5. The Teleportation Experiment

New insight on the dynamics of spatial representations in CA3 has recently come from work by Jezek et al. [73]. The aim of this study was to investigate the time course of map replacement, by determining the evolution of neuronal activity in response to an abrupt transition between two environments, resulting from a sudden change of the external cues used to define each spatial context. The recordings were performed in CA3. 

### 5.1. The Paradigm

Rats were trained in two different text boxes, designated A and B, which differed only in the type of illumination. The rats spent several days exploring these environments. The paradigm was intended to induce global remapping of the activity in CA3, by generating two uncorrelated representations for the two environments. The test phase took place after such uncorrelated representations had stabilized in the training phase. The rat was started in one of the two familiar environments, then after some time the rat was teleported to the other environment: the lights were abruptly switched to those of the other environment, taking the rat instantaneously, as it were, from one place to another.

### 5.2. Observations

A number of analyses [73] indicate that even during these extremely rapid transitions between reference frames, the activity in the hippocampus is determined by attractor dynamics. The representation expressed by CA3 at any time is almost always one corresponding to one of the environments—few intermediate representations are observed, corresponding to mixtures of the two representations for box A and B, with elements from them both. The competition between the representations, stimulated by the fast transition, is revealed by the appearance, just after the teleportation, of rapid switches from one representation to the other, a phenomenon labeled by the authors as “flickering.” One peculiarity of such flickering is its relation to theta oscillations. The reexpression of one of the two representations is generally completed within the time span defined by a theta cycle. The theta rhythm seems to regulate the timing of the dynamics also by marking the alternation between the two representations.

These results seem to indicate a major role of the theta oscillations in regulating attractor dynamics in the CA3 network. The propagation of activity through recurrent collaterals appears to be controlled by the periodical modulation of the physiological variables in the network. 

Although this phenomenon seems related to the Hasselmo hypothesis of a role of theta oscillations in segregating encoding from retrieval, its raison d'être might be different: to allow a trial-and-error-like procedure. Repeated convergence to attractor states might offer the rodent the opportunity to self-correct, a possibility that can be very useful under conditions where input cues are ambiguous, weak, and confusing. In the case of this experiment, the conflict is between the external cues, characterizing the final box, and cues coming from path integration which, because of the very fast transition, still refer to the initial box. The possibility to continuously integrate clues about location and to formulate distinct hypotheses within a limited time may be important in order to properly adjust behaviour to the external world. 

## 6. Our Model for Teleportation 

To test the feasibility of an involvement of the theta rhythm in the dynamics of memory retrieval and to check that the phenomena observed in the teleportation experiment can be ascribed to these oscillations, we have tried to simulate the results with a simple model representing the CA3 region of the hippocampus.

The goal of these simulations is to see whether the flickering events may arise from a modulation of the activity in the presence of partial and ambiguous external inputs. We thus compare the activity in the network in the presence of theta oscillations and without them, when all other conditions are the same. Even if the neural model is intended to represent the CA3 region of the hippocampus and its dynamics, the structure of the network and its operating parameters do not exactly reproduce those in the real brain. To focus on the effects of theta modulation on network performance and to reduce any other spurious effect coming from uncontrolled features of the model, not relevant for the present issue, we have tried to reduce the complexity of the simulations as much as possible. For the sake of clarity the model then represents a system only similar to that present in the brain, retaining—it is hoped—the characteristics important for the problem at hand. 

### 6.1. The Model

We first construct the representations of the two environments. They are two geometrically identical square boxes of size *S*. The network is comprised of *N* units. In the standard condition, the two environments are represented by two separate populations of units, half active in environment A, the other half in environment B. Each unit has a single place field in the assigned environment. Its firing rate around the place field center is defined by a Gaussian function. Place field centers are regularly spaced, arranged on a grid covering the whole environment. The two representations are therefore identical and differ only in the subset of units which are active. The Gaussian bumps representing the place fields are defined as all having the same effective size and height. The network is fully connected, and the strength of the connections is prewired. The structure of the connections is functional to the establishment of a quasicontinuous attractor surface, expressed by synaptic weights that follow an exponential decreasing function of the distance between place field centers. The weight of the connection between cell *i* and *j* is then written as

(1) $$\begin{array}{c} W_{\text{rec}}^{ij}=C*\exp\;\;\left(\frac{-d_{ij}}{S}\right) \end{array}$$

if the two cells have place fields in the same environment, while it is set to zero if they belong to different environments.

The model is a firing rate model and the neurons are simple threshold linear units. The activity is thus governed by the following equation:

(2) $$r_{i}\left(x;t_{n}\right)=g{\left\lfloor \mathrm{Ext}\;\left(x\right)+\sum_{j}W_{\text{rec}}^{ij}r_{j}\left(t_{n-1}\right)-T\right\rfloor }_{+}.$$

For each cell, the activating current is comprised of two terms: an external input and the recurrent collateral contribution. The gain *g* and the threshold term *T* are modified to regulate the average activity and the sparsity of the network.

### 6.2. Generating the Templates

The simulation consists of two phases. First, we generate a trajectory for the virtual rat. The simulation is continuous in space but discrete in time. In each time step, intended to correspond to roughly 15 ms, the virtual rat moves by 0.5 cm in a direction similar to the direction of the previous time step, with a small amount of noise. At each time step we determine the input activity associated with the position occupied by the rat. To that effect, movements are considered to occur along the same trajectory in both environments, and so inputs are updated for both environments. The activity of each unit is determined according to (2), with the external input set according to the location in the current environment; while the term representing the activation induced by the contribution of the recurrent collaterals depends on the activity of the network calculated at the last step. This procedure emulates the activity that is elicited in CA3 during navigation in each environment, providing the templates of the spatial representation of the two environments. To study the effects of teleportation on the dynamics of CA3, such templates are then compared with the state the network takes when, instead of a full cue about one environment, it receives conflicting inputs about both.

### 6.3. Test Phase

The second part of the simulation reproduces the teleportation experiment. The same path is followed as for building the templates, so that the rat runs through the same trajectory. We then divide the path into three sections. In the first part, we fed the network with a full cue about environment A. Then, we teleport the rat to environment B. The external cue is suddenly changed. To simulate the ambiguity and the conflict between external cues and internal path integration, we modify the input to represent this confusion. While before we fed the network with a full cue, we now reduce it to 20%, in that only a subset of the cells now receives external input. Moreover, the cue does not represent a single environment: a fraction refers to environment A and the rest to environment B, which means that both units coding for A and for B receive external input. Time steps are grouped in sets of eight, which then represent eight phases within individual theta cycles, when we introduce theta modulation. During each set/cycle the cue is kept constant, in the sense that, while the input changes as the animal moves and changes location, the units that receive external input remain the same. At the beginning of each cycle these cells are selected with a certain probability, representing the proportion of environment A and B in the external cue, which changes at each cycle. While the number of neurons receiving an external input is kept constant, the amount of activated neurons in each group has a 25% variability. This state of confusion is held for a certain time, 10 sets or theta cycles in our simulations. At the end of this phase, we turn the external input to a full representation of environment B. The network is then fed a full cue corresponding to the second environment, simulating a rat that has finally understood which box it is in. 

All the simulations are first run without any periodic modulation of the activity in the network. We then introduce theta oscillations by periodically modulating the mean activity and the sparsity of the population, with a period of eight time steps [74]. We also modulate the strength of the external cue in a similar way, by varying both the number of units receiving external input and the intensity of the stimulation. We vary the amplitude of this modulation and the relative phase Δ between the oscillations of the network and of the external input. 

**Figure 3:**
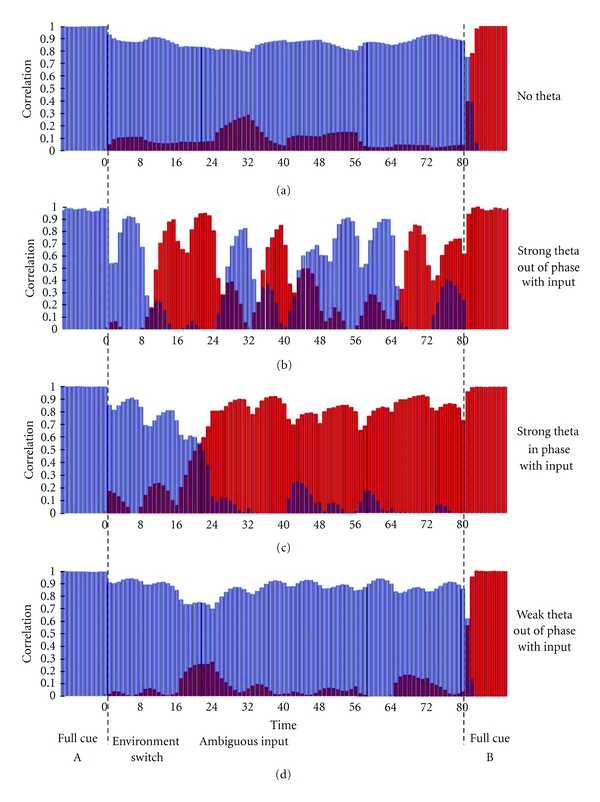
Results of our simulation of the teleportation experiment. Bars represent correlation with the two different representations at each time step. Different plots are relative to different conditions: “strong” is 80% variation in firing rate and sparsity between the peak and the through and “weak” is 20% variation; in phase means Δ = 0° and out of phase means Δ = 180°.

In Figure 3 we plot the correlation between the activity generated during this test phase and the activity obtained during normal exploration, in the same location. 

### 6.4. Results

As expected, the network with no modulation generally fails to respond to changes in the external environment. After the first switch, the action of the recurrent collaterals is to maintain the network in the attractor relative to environment A, even if the external input has changed. The external cue can produce an effective change in the dynamics of the network only when very strong. At the end of the simulation, with the full cue of environment B, the system actually follows it and retrieves the other representation. In general, however, in this range of parameters, the dynamics of the network do not respond rapidly to changes in the external environment. 

Flickering events appear, instead, when we introduce strong theta oscillations and in antiphase with the modulation of the input. Such rapid transitions between the retrieval of one attractor and the retrieval of the other, in the time span of a theta cycle, are similar to those observed in the experiment of Jezek et al. [73] and appear with the first switch between the environments. The simulations show the effect of modulating the activity in enhancing the flexibility of the network and its ability to respond rapidly and accurately to changes in the input. Note that, while reacting to the changing external cue, the network does not simply reproduce the ambiguity of the input. In each theta cycle, with a few exceptions, the system retrieves one of the two representations and discards the other. At every theta cycle this retrieval appears to be as good as the one obtained in the case with no oscillations. These effects disappear when oscillations are smaller, or when the input and the network oscillate in phase. 

## 7. Discussion

Our model suggests that modulating the activity of the CA3 region of the hippocampus may have significant effects on memory processes, but only if the modulation is strong. In the model, flickering events as described by Jezek et al. [73] in their teleportation experiment appear, but only in the presence of strong theta modulation. This is consistent with an active role of theta activity in stimulating fast transitions between attractor states [75]. 

### 7.1. The Conflict between Attractor Dynamics and External Input

During memory retrieval, the activity generated by an external stimulus is propagated through the collaterals connecting the units of an associative memory network. Since memories are stored by modifying the strength of these connections, this propagation leads to an activation of the network which corresponds to one of the stored memories—what we call an attractor. Which one of the multiple memories stored in the network will be retrieved depends on the characteristics of the initial external input. Such associative memory networks function optimally when the external input serves only as a cue and neuronal activity is driven maximally by the recurrent connections. The internal dynamics of the network should be relatively unconstrained by the external input, to allow for convergence to the attractor state, even in the presence of an incomplete or distorted cue. 

The drawback of this operating regime is that the network loses in flexibility. The network is likely to remain in an attractor once it has reached one, and it needs a very strong shove to leave it. It is not reactive to external inputs unless this input is strong and significantly different from its current state. In our case, in which the external input is a mixture of two different representations, the action of the recurrent collaterals is to let the system choose between one of them, generally the stronger of the two, and it drives the activity of the neurons towards the corresponding attractor. But as the external input changes, if neither representation clearly prevails, it is very difficult for the network to modify its state, at least, that is, when no modulation is present.

### 7.2. Theta Oscillations as a Regulator of External Input Dominance

The effect of introducing a modulation of the activity and of regulating accordingly the firing rates of the neurons is to control the relative strength of the inputs each neuron receives, the external cue, and the recurrent reverberation. As the number of active units and the level of global activation changes, even if the strength of the connections remains unvaried, the driving force of the system is shifted from external to internal inputs and vice versa. When only few cells are active, those which receive a strong external input have a major advantage in the competition and therefore are more likely to pass the inhibition threshold and to fire. They in turn activate new units as inhibition gets weaker. As new units are activated through the current injected in the collaterals, the activity in the network evolves, driven by the internal dynamics generated by the initial pool of active cells. If cells representing both maps are activated, then as the threshold is lowered there is a rush to recruit new units, in a competition between the two representations. When the inhibition reaches its minimum, one of the two eventually takes over, and the system falls in one of the two attractors. What happens next is crucial for the appearance of flickers. Once the inhibition rises again, the active population starts to shrink. In the beginning the collaterals continue to determine the active units, but as the inhibition approaches its maximum again, the contribution of the external input becomes critical. In a regime of very strong competition, the activity of the system is strongly related to the input that it receives at that time. As before, this small core of units is then responsible for the activation in the next theta period, while the state the system was in is forgotten. The system goes through a sort of bottleneck that allows resetting the state of the units and forcing the system to discard the information conveyed by the recurrent collaterals. At each onset of the cycle the internal dynamics of the system are thus almost independent of the past activity. The system is potentially able to select a new attractor at each theta cycle. 

**Figure 4:**
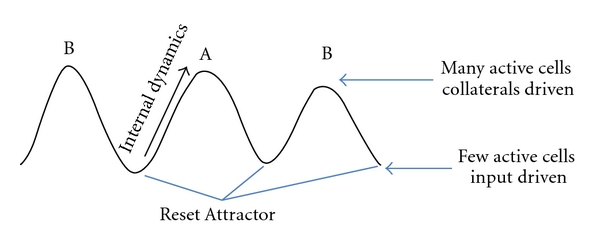
Pictorial representation of the dynamics in our model.

**Figure 5:**
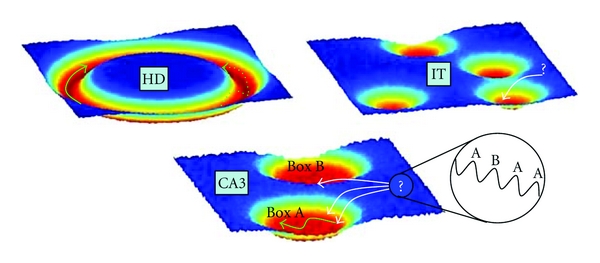
Different types of (idealized) attractors suggest distinct interpretations of neural dynamics observed in experiments. In the 1D ring attractor putatively underlying the head direction system [77] (top left), changing head direction implies moving, whether continuously (green arrow) or in jumps (dashed arrow), along the attractor. Convergent neural activity in IT [76] (top right) and rapid flickers in CA3 [73] (bottom) can be interpreted as rolling down into the attractor basin (white arrows). In CA3, spatial representation activity can also move along the flat attractor valley [79] (green arrow).

Attractor dynamics is a theoretically attractive concept, and attempts have been made to apply it to explain neural dynamics in a range of experimental situations, as in the schemes of Figure 4. The more classical notion of a discrete, point-like attractor has been used to interpret convergent neural activity in monkey inferotemporal cortex (IT) [76]. Its conceptual extension, the continuous attractor, can been applied instead to interpret recordings of head direction cells [77] and place cells in single environments [78, 79], which may reveal movements along the nearly flat “bottom valley” of a continuous attractor. Although quite different from each other, these dynamics are also quite different from those observed in the teleportation experiment, which seem to reflect the presence of periodic modulation. In the nonmodulated condition, either the system is driven by external input, with little room for attractor dynamics, which merely serves to keep it within the correct valley [77–79], or, if inputs subside, it is capable of memory retrieval, but rigid and unable to rapidly adapt to further external changes [76]. Introducing theta modulation allows implementing both these regimes in the same system, by segregating them to different theta phases. The network is input driven in the presence of strong inhibition and then progressively switches to be driven by recurrent connections as the theta period progresses. In this perspective, the addition of theta oscillations, while not essential, provides a new twist in an already consolidated schema. Flickers could be the result of successive descents in different attractor basins, while the internal attractor dynamics, taking place along the continuous attractor representing each environment, may remain unaffected by the modulation (Figure 5).

### 7.3. Strong Modulation …

Our simulations indicate that to obtain this particular effect the amplitude of the theta oscillation is crucial. The difference between the number of active units and the average firing rate at the peak and at the trough of the theta cannot be too small. Of course this conclusion is limited by the particular assumptions and simplifications we made for our model. The way we implemented theta effects on the system is simple and most straightforward. We do not explicitly model the inhibitory neurons present in the hippocampus, nor we take into account complex interactions between them and the pyramidal neurons. The effects of inhibition are modeled as a homogeneous regulation of the gain and of the threshold applied to the firing rate of all the neurons. Actually, as we have already said, there is growing evidence on the complex dynamics linking the excitatory and the inhibitory populations in the hippocampus. The different phase locking exhibited by distinct inhibitory neuron types and the fact that this differentiation roughly corresponds to the classification in dendritic or somatic interneurons suggest the possibility that such articulation is functional to an inhibitory mechanism not described in detail by our model [80, 81]. Moreover, the particular feedback inhibition microcircuitry present in the hippocampus, which regulates local activity and is not detailed in our model, may be another source of complex effects not expressed in our simulations [82–91]. Input-specific suppression and other nonlinearities, which may arise from the incorporation of this finer structure in a model, may extend our results also to the case of gentler oscillations.

### 7.4. … And out of Phase

Another indication that emerges from our model is the role of the timing of the activity in different elements of the hippocampal circuit. As the simulations show, the relative phase of the oscillations of the incoming input is important to maximize the effects of the theta modulation on the dynamics of our recurrent network. It appears that the hypothesized mechanism functions optimally when the two oscillations are out of phase. This makes sense, as such a phase relation of the two oscillators enhances the separation between the two regimes of input dominance and recurrent dominance, which is at the base of our model. A concurrent rise and fall of the activity in the network and in the strength of the input actually cancels the benefits obtained with the introduction of the modulation. The input then generates interference with the internal activity when the dynamics of the system should be driven by the latter, and it is weak when its turn comes to lead. On the contrary, an external input which is in antiphase with the modulation of the network can act effectively during periods of low activity and then gently bow out when the network retrieves the attractor. In conclusion, our data better reproduce experimental observations when the recurrent network is driven by an external input which oscillates in magnitude with the same period but a different phase.

### 7.5. Some Remarks on Information Flow in the Hippocampus

This leads to some comments about the operation of the real hippocampus. As reviewed above, the phase relations of the activity of the different regions of the hippocampus and of the neighboring afferent regions of the entorhinal cortex are rather articulated.

Consider the CA3 region. The two external inputs this region receives have specific and different theta phase modulation of the firing rate [17]. The neurons in the DG, which projects to CA3 through the mossy fibers, fire almost in conjunction with cells in CA3, and moreover the pattern of activation is very similar: the activity in the two regions spreads and then shrinks in almost perfect synchrony [19]. On the other hand, activity in the EC layer II, which projects to CA3 through the perforant path, appears to be maximal at a completely different theta phase [9, 92]. Between the two bumps of activity, the one in EC and the one in CA3, there is approximately a quarter of a theta cycle [17].

This configuration seems to replicate the one described by our model. If we want to infer from the simulations, we should conclude that during the teleportation experiment, just after the switch of environments has occurred, the recorded activity in CA3 is mainly due to stimuli coming from the entorhinal cortex, in essence bypassing the DG. This conclusion suggests a revision of the prevailing view of information flow in the hippocampus in normal conditions. The normal path of activation, involving in the order layer II of entorhinal cortex, DG, CA3 and finally CA1, may indeed be the “normal” one, but during encoding, or when inputs are strong. During retrieval or when inputs are weak, there is some evidence that CA3 may operate in different and alternative regimes.

- First, behavioral experiments on spatial learning in rodents indicate that DG inactivation and DG lesions greatly impair the acquisition of new memories while sparing the retention of already stored ones [93–95]. These studies indicate that the combined action of perforant path and recurrent collaterals is sufficient to perform retrieval of stored memories. So, while minor during robust DG activation, the contribution of the perforant path emerges when left alone. Synaptic plasticity acting on the perforant path allows associating the activity of the entorhinal cortex with the representation imposed by dentate activity, thus enabling future pattern retrieval.
- This alternative route, which forfeits the contribution of the DG, appears to contribute to another phenomenon observed in the hippocampus, the so-called sharp wave ripples (SWRs). Indeed, this physiological pattern of activity originates directly in CA3, triggered by synchronized activation of pyramidal cells, and does not involve at all the dentate gyrus [96, 97]. It is plausible to suppose that these sudden bursts of activity may be initiated by a weak input arriving from the entorhinal cortex [10, 98]. The activation of even a small subset of CA3 neurons could initiate a cascade of excitation across the recurrent collaterals, leading to reverberating activity that eventually spreads to downstream regions like CA1 [99, 100] (possibly with a more active and independent role of the latter in processing this signal than initially thought, as recent findings show that the phenomenology of the CA1 ripples is too complex to be a mere reproduction of CA3 activity during these episodes [65, 101]).

It is possible that inhibitory dynamics similar to that underlying SWR generation [53, 79, 80] may operate in the rat hippocampus during the interval following the teleportation. The confusing and misleading context may limit the activity in the EC to a fraction of that normally observed. In this case, the input originating from this region may be insufficient to properly activate the corresponding spatial representation in the DG. Still it can be enough to generate a cascade of activity in CA3. The presence of recurrent connections may act as an amplifier of an otherwise weak signal. 

The concomitance of maximal DG and CA3 activation is in accordance with the generic idea that phase synchronization is functional to optimize neural communication and synaptic plasticity [5]. Having the neurons in the two regions active at the same time can be used to maximize the information transfer between the two regions during the storage of new representations, even if we cannot exclude that this layout may be partially modified by specific effects arising from the particular structure of the feedforward inhibition of DG on CA3 [86]. 

On the other hand, the prevailing phase difference observed in the firing of the excitatory populations in EC and CA3 may result in an effect not captured by the generic idea. This particular timing allows the dynamics of CA3 activity to select a new attractor at each theta cycle. Of course if the external cue, even if partial, is correlated with a single stored memory, the system will simply retrieve the corresponding attractor. This mechanism becomes interesting when the cue is ambiguous and is comprised of a mixture of different representations. What happens then is that the choice of the attractor may amplify a bias or even a statistical fluctuation of the composite cue. If we observe the choices the system makes over a certain period, the number of times it chooses a given memory could be related to the fraction of the cue representing that particular memory. Moreover, because the choices are distributed in time, this mechanism may allow integrating information arriving at the hippocampus at different moments. Faced with a changing and unstable input, multiple choices provide statistical information about the environment and allow the system to correct an occasional wrong choice. If the network proceeds three times towards the attractor corresponding to A and only once towards B, it can reasonably concluded that the box is A, even if the cue was ambiguous. The freedom left to the system, from the external input, and the resetting of the state of the system at every cycle, both obtained with the introduction of the theta oscillation, are the elements generating flickering after teleportation, in our model. 

## 8. Conclusions

Theta oscillations seem to satisfy a fundamental need for organization. Their appealing regularity stands against a background of noisy and complex ensemble activity by single units. It allows bringing back the continuous flow of brain activity into more reassuring frames and describing neuronal dynamics in terms of periods and phases, with different properties according to the functions we want to assign them. 

**Figure 6:**
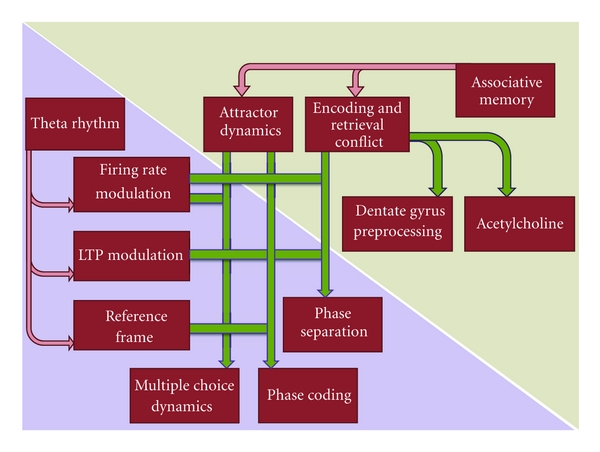
Overview of the theories reviewed here and their conceptual relations.

Rather than considering the theta rhythm as a product of neuronal activity, one may consider a perspective in which the factors are inverted, and the theta rhythm drives the neurons. The two options may be difficult to disentangle, as with any purportedly causal dependence in the brain. This approach, in any case, has produced theories assigning to the theta rhythm important roles in neural computation (Figure 6). In the rodent hippocampus, where these oscillations are most prominent, this intellectual trend is particularly lively. 

Theta oscillations have been proposed to regulate the internal flow of information among the various subregions of the hippocampus or to modulate some important physiological parameters, like the strength of induced synaptic LTP. Or else, they have been proposed to generate a neural code of their own, beside the firing rate code, based on the relative position of individual spikes.

The effects of theta modulation may however be subtler, and only relevant in particular conditions. The phase precession phenomenon, for example, can be interpreted as resulting from the interaction, in the rodent hippocampus, of theta rhythmicity with attractor dynamics, rather than as revealing a sophisticated phase coding of position [102]. Likewise, the mechanism described here may be regarded as another “unintended,” or perhaps just additional, feature of superimposing theta modulation onto attractor dynamics. Without the pretension of being exhaustive or conclusive, our simulations suggest that the introduction of theta oscillations generates significant effects only if they are particularly strong and have particular phase relations, conditions that are not always satisfied in the hippocampus. Even if they are not capturing the whole complexity of the hippocampal network, by far, these simulations at least indicate that these parameters have to be evaluated carefully and that the application of theta-based theories requires quantitative assessment. Specifically, the model suggests that the relevant memory operations can be independent of the theta rhythm, but that its presence may be important in optimizing and refining them. The teleportation paradigm, with the emergence of flickering, the rapid alternation between representations, may give us some insight into these issues and help us to unravel the finer structure of memory dynamics in the hippocampus, possibly also clarifying the role of theta oscillations.
